# An Update on Neurotrophins

**DOI:** 10.2174/157015911798376280

**Published:** 2011-12

**Authors:** Célia Duarte Cruz

Neurotrophins are important proteins that regulate survival, development and function of neurons. They are produced by a variety of cells and exert their effects upon binding to specific tyrosine kinase receptors. The first neurotrophin to be identified was Nerve Growth Factor (NGF). From the pioneer studies performed by Rita-Levi Montalcini, awarded with the Nobel Prize, other neurotrophins have been identified and this family of neurotrophic factors now includes Brain Derived Neurotrophic Factor (BDNF), Neurotrophin 3 and Neurotrophin 4. Their function as pro-survival proteins has been consolidated but subsequent studies have demonstrated that neurotrophins may also play a role in some pathological conditions. This is the focus of this special issue of Current Neuropharmacology. 

The paper by Allen *et al.*, addresses the involvement of neurotrophins, NGF and BDNF in particular, in Alzheimer’s disease and the possibility of using neurotrophin-based therapies.

Frias *et al.*, summarize studies that support the involvement of neurotrophins in the lower urinary tract (LUT) dysfunction and suggest the use of urinary neurotrophins as biomarkers of LUT pathologies.

The paper by Neto *et al.*, addresses the involvement of neurotrophins in depression, a poorly understood pathology with an increasing prevalence in Western countries.

The paper by Siniscalco *et al.*, addresses the involvement of neurotrophins in neuropathic pain, a complex pathology difficult to tackle with, and its importance as targets for neuropathic pain treatment. 

Teng *et al.*, summarize the clinical uses of stem cell-based administration of neurotrophins for the treatment of spinal cord injury.

This issue is published in memory of David Dawbarn, who passed away unexpectedly in January 2010. David dedicated most of his work at the Bristol University to understanding the importance of neurotrophic factors in Alzheimer’s disease, unraveling the molecular interactions between neurotrophins and their high-affinity receptors. His research was a key factor to the design of new therapies for the treatment of Alzheimer’s disease, pain and asthma. He is greatly missed by the scientific community, friends and family. 

